# Er^3+^-doped transparent glass ceramics containing micron-sized SrF_2_ crystals for 2.7 μm emissions

**DOI:** 10.1038/srep29873

**Published:** 2016-07-19

**Authors:** Yiguang Jiang, Jintai Fan, Benxue Jiang, Xiaojian Mao, Junzhou Tang, Yinsheng Xu, Shixun Dai, Long Zhang

**Affiliations:** 1 Key Laboratory of Materials for High Power Laser, Shanghai Institute of Optics and Fine Mechanics, CAS, Shanghai 201800, China; 2 University of Chinese Academy of Sciences, Beijing 100049, China; 3 Key Laboratory of Photoelectric Materials and Devices of Zhejiang Province, Ningbo, 315211, China

## Abstract

Er^3+^-doped transparent glass ceramics containing micron-sized SrF_2_ crystals were obtained by direct liquid-phase sintering of a mixture of SrF_2_ powders and precursor glass powders at 820 °C for 15 min. The appearance and microstructural evolution of the SrF_2_ crystals in the resulting glass ceramics were investigated using X-ray diffraction, field-emission scanning electron microscopy and transmission microscopy. The SrF_2_ crystals are ~15 μm in size and are uniformly distributed throughout the fluorophosphate glass matrix. The glass ceramics achieve an average transmittance of 75% in the visible region and more than 85% in the near-IR region. The high transmittance of the glass ceramics results from matching the refractive index of the SrF_2_ with that of the precursor glass. Energy dispersive spectroscopy, photoluminescence spectra, and photoluminescence lifetimes verified the incorporation of Er^3+^ into the micron-sized SrF_2_ crystals. Intense 2.7 μm emissions due to the ^4^I_11/2_ → ^4^I_13/2_ transition were observed upon excitation at 980 nm using a laser diode. The maximum value of the emission cross section of Er^3+^ around 2.7 μm is more than 1.2 × 10^−20^ cm^2^, which indicates the potential of using transparent glass ceramics containing micron-sized SrF_2_ crystals for efficient 2.7 μm lasers and amplifiers.

Recently, Er^3+^ ions have been widely investigated and used in the field of optics as active optical ions because of their unique luminescence properties and low-cost, high-efficiency and commercially available the pump resources^1^,^2^,^3^,^4^,^5^,^6^,^7^,^8^. In particular, Er^3+^ ions can easily achieve population inversion and amplification around 2.7 μm because of the narrow energy gap (~3600 cm^−1^) between ^4^I_11/2_ and ^4^I_13/2_^9^. This radiation is interesting for many medical and spectroscopic applications^10^, optical communication^2^, and as an excitation source for infrared (IR) optical parametric oscillators^9^. However, the efficiency of the 2.7 μm emissions is strongly dependent on the phonon vibration of the medium because the mid-infrared (2.6–2.9 μm) fluorescence will be quenched in a high phonon-energy environment^11^. Therefore, an appropriate host material is extremely crucial for achieving high efficiency mid-infrared emissions.

Mid-infrared host materials are mainly low-phonon-energy fluoride materials, such as fluoride glasses and fluoride single crystals. Among these host materials, Er^3+^-doped fluoride glasses have achieved efficient 2.7 μm lasing emissions, but exhibit inferior thermal and mechanical properties^6^,^12^. Er^3+^-doped fluoride single crystals are considered ideal host materials for Er^3+^ ions while retaining their excellent 2.7 μm emission performance because of their low phonon energy, high thermal conductivity, and excellent heat shock resistance^9^. Unfortunately, the strict requirements for fabricating large, homogenously doped, and high-quality fluoride single crystals is a serious obstacle that hinders the crystal-based mid-infrared applications^9^,^13^.

Transparent glass ceramics (GCs), which possess the combined properties of glasses and crystals, are promising optical materials that compete with single crystals and glasses. Meanwhile, many experimental studies have confirmed that rare-earth (RE) ions are selectively embedded in the fluoride crystals distributed in the glass matrix. Therefore, Er^3+^-doped GCs containing fluoride crystals are expected to achieve highly efficient mid-infrared emissions. Wei^6^ and Wu^12^ have successfully observed efficient 2.7 μm emissions in Er^3+^-doped NaYF_4_ and CaF_2_ nanocrystals embedded oxyfluoride GCs prepared by melt-quenching method, respectively. As is well known, some disadvantages of melt-quenching method, such as high preparation temperature and energy consumption, uncontrollable crystal formation, and long and complex heat treatment, severely hinder the GCs-based applications^14^,^15^,^16^. On the other hand, the optical properties in these GCs are achieved only through a careful partial crystallization of the nanometer scale grains. However, network modifiers and impurities in the glass matrix tend to accumulate on the surface of the nanocrystals because of the high surface energy, which may change the properties of the nanocrystals^17^. Meanwhile, defect energy levels are easily introduced into the forbidden band of nanocrystals because of large surface defects, resulting in defect luminescence^18^. Large grains can prevent the problems encountered in nanocrystals because of a small surface-to-volume ratio. Fan *et al*.^9^ obtained intense IR fluorescence around 2.7 μm in Er^3+^-doped transparent GC containing large CaF_2_ crystals by a technique similar to the liquid-phase-sintering method, which implies that Er^3+^-doped transparent GCs containing large fluoride grains exhibit a high potential for mid-infrared applications. Additionally, abovementioned method possesses low preparation temperature and precise crystal phase control that compete with melt-quenching method. Unfortunately, a complex and time-consuming preparation process including coprecipitation, ball-milling and highly precise temperature and time control is requirement. Most importantly, long-time sintering is necessary for abovementioned method to remove pores and achieve the growth of crystals, which causes strong glass components volatilization. The changes of glass components will increase the differences in the refractive index between crystalline phase and glass phase and scattering losses. Meanwhile, glass components volatilization entails a certain degree of randomness, which is difficult to accurately adjust the refractive index of glass matrix. Measures must be taken to address these problems.

In order to avoid the difficulties of^6^,^9^,^12^, a liquid-phase sintering route was designed by tuning components of PG and optimizing the preparation process in foundation of existing technique^9^,^19^, which is directly sintering a mixture of commercial fluoride powders and PG powders prepared by grinding at low temperature for a short time. This liquid-phase-sintering route has several advantages over existing GCs preparation method such as: precise control over the crystal phase and refractive index, short preparation time, low preparation temperature and energy consumption, and simple and low-cost synthetic approaches, which results in a high financial and energetic saving.

In this study, based on remarkable optical performances and high potential for mid-infrared applications of SrF_2_ crystal and GCs containing SrF_2_ crystals^20^,^21^,^22^, Er^3+^-doped transparent GCs containing micron-sized SrF_2_ crystals were fabricated by abovementioned liquid-phase sintering route and characterized. To the best of our knowledge, we obtained, for the first time, transparent GCs containing micron-sized SrF_2_ crystals and 2.7 μm emissions in GCs containing SrF_2_. Intense 2.7 μm emissions generated by GCs have been discussed in detail for possible applications in mid-infrared lasers and amplifiers.

## Experimental

### Sample preparation

Fluorophosphate (FP) glass, which is a promising laser material, has remarkable spectral properties, good chemical and mechanical stability, low OH^–^ content, and a low melting temperature^23^. In particular, the refractive index of FP glass can be readily tuned to match that of SrF_2_, yielding highly transparent GCs containing large SrF_2_ crystals. In our experiment, Er^3+^ doped FP glass with a nominal molar composition of 35CaF_2_-18MgF_2_-10SrF_2_-18BaF_2_ -3NaF-6AlF_3_- 7Na(PO_3_)_3_- 2.7Al(PO_3_)_3_ -0.3ErF_3_, denoted as precursor glass (PG), was prepared by the conventional melting-quenching technique. The refractive index of FP glass was matched with that of the SrF_2_ crystal using the GE-SYSTEM software. Samples were prepared using analytical-grade NaF, MF_2_ (M:Ca, Sr, Ba), AlF_3_, ErF_3_, Na(PO_3_)_2_ and Al(PO_3_)_3_ by the liquid-phase sintering method. A GC with a composition of 45SrF_2_-55 PG (wt%) was prepared from SrF_2_ and PG powders. SrF_2_ powders (4.5 g) were blended directly with PG powders (5.5 g) by grinding in an agate mortar for 5 min. The mixture was melted in an electric furnace at 820 °C for 15 min. The melts were then quenched in stainless steel molds and pressed into thin plates. The samples were annealed at 380 °C (Tg = 392 °C) for 2 h to reduce internal stress, when Er^3+^:SrF_2_ GCs were obtained.

### Sample characterization

The crystallization behavior of the samples was investigated using a Philips PANalytical X’Pert X-ray diffraction (XRD) system at 40 kV and 40 mA with a Cu target X-ray tube. The transmittance characteristics were investigated by a Lambda 1050 UV/VIS/NIR spectrophotometer (Perkin Elmer) for wavelengths ranging from 300 to 3000 nm. The thermal behavior of the PG was characterized using thermogravimetry (TG) and differential thermal analysis (DTA) at a heating rate of 5 K/min in air from 100 °C to 1000 °C using an EXSTAR SII:TG/DTA7300 from Japan. The refractive index of the samples was obtained by the prism minimum deviation method using a WYV V-Prism refractometer (CANY, Shanghai, China) with an accuracy of n_D_ ± 5 × 10^−5^. The microstructural evolution of the samples was studied by a field-emission scanning electron microscope (FESEM, HitachiS-4800, Japan) equipped with an energy-dispersive X-ray spectrometer (EDS) and transmission microscope (Olympus). The emission spectrum with excitation of a diode laser at 980 nm was recorded using by a PMT detector (R928) at 25 °C.

## Results and Discussion

**Figure 1:**
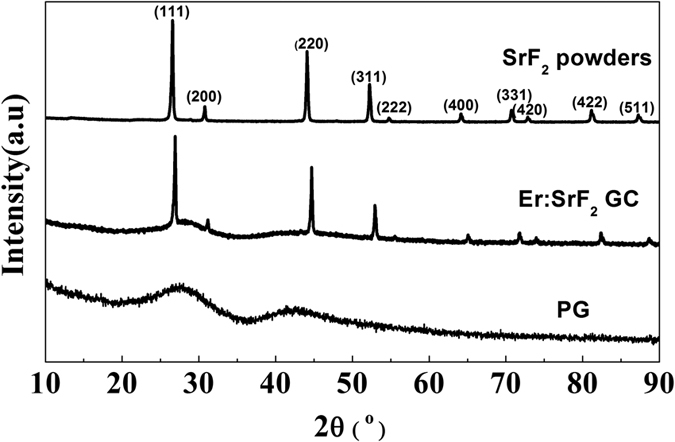
XRD pattern of Er^3+^-doped PG, Er^3+^-doped GC and SrF_2_ powders.

XRD measurements were performed to examine the crystallization behavior. Fig. 1 shows the XRD pattern of the Er^3+^-doped PG, Er^3+^-doped GC, and SrF_2_ powders, respectively. Only two broad humps are observed in the XRD pattern of the PG, indicating that no spontaneous crystallization occurs in PG. After liquid-phase sintering at 820 °C for 15 min, two broad envelopes of glass diffraction and some intense sharp peaks of the crystalline SrF_2_ cubic phase emerge in the XRD curve of GC, which confirms that SrF_2_ crystals were successfully incorporated into PG. Interestingly, a small right shift of the diffraction peaks is observed in the GC compared to the SrF_2_ powders. This is attributed to a decrease in the interplanar spacing (d) according to the Bragg equation, which implies ion doping in SrF_2_ crystals. Notably, the diffraction peaks of GCs are sharper than the peaks for the SrF_2_ powders, indicating that growth of crystalline SrF_2_ occurred during the preparation process.

**Figure 2:**
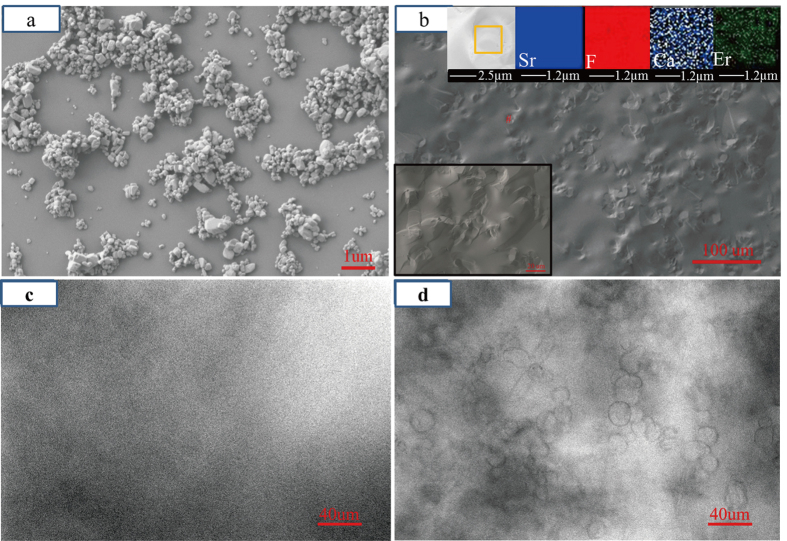
SEM image of the SrF_2_ powders. (**a**), SEM image of the fractured surface of GC (**b**) with the associated Sr (blue), F (red), Ca (blue-white), and Er (green) elemental mappings in the insets. The inset in the lower left of (**b**) shows the magnified SEM image of the fractured surface of the GC. The transmission microscope micrograph of the interior of the PG (**c**) and GC (**d**).

SEM images showing the morphology of the SrF_2_ powders and the fractured surface of the Er^3+^-doped GC containing SrF_2_ are shown in Fig. 2(a,b), respectively. As shown in Fig. 2(a), the average size of the SrF_2_ powders is ~300 nm. In Fig. 2(b), we can see that the fractured surface of the GC is rough, and large amounts of isolated particles are embedded in the glass matrix. No pores are observed in the magnified SEM image (Fig. 2(b), inset, lower left), which indicates that PG has the ability to remove pores. Pores can be easily removed by dissolution in the liquid glass phase and outward diffusion to the environment because of the low viscosity of FP glass. Based on the XRD pattern shown in Fig. 1, these isolated particles are considered as SrF_2_ crystals. To confirm this conclusion, elemental mapping was used to discern these isolated particles, as presented in Fig. 2(b). The analysis shows that Sr, F, Ca and Er signals are detected in the isolated particle of GC, whereas the presence of Sr and F is stronger than other elements. This result indicates that the isolated particle is SrF_2_ and that Er^3+^ ions together with Ca^2+^ ions are incorporated into the SrF_2_ crystal lattice. The extra Ca is attributed to ion exchange during liquid-phase sintering. This observation supports the abovementioned doping assumption. The substitution of the larger Sr^2+^ (1.12 Å radius) with the smaller Ca^2+^ (1.00 Å radius) and Er^3+^ (1.00 Å radius) during the preparation process shrinks the crystal lattice. As a result, the interplanar spacing of SrF_2_ decreases, as shown in Fig. 1. Some information on the quality of the isolated particles, such as size and distribution, can be obtained by direct imaging using the transmission microscope. Fig. 2 (d) displays the transmission microscope micrograph of the interior of the GC. The nearly spherical particles observed in the transmission micrograph correspond to SrF_2_, as identified by the XRD and EDS results. It can be seen that many SrF_2_ particles that are ~15 μm in size are randomly distributed in the glass matrix. For comparison, the transmission microscope micrograph of PG is shown in Fig. 2(c). There is no SrF_2_ phase and only the glass phase is observed. Note that the size of the SrF_2_ particles inside the GC is a few tens of micrometers, while that of the SrF_2_ powders is hundreds of nanometers; this result is consistent with that of XRD pattern analysis which shows the growth of the SrF_2_ crystals during liquid-phase sintering.

**Figure 3:**
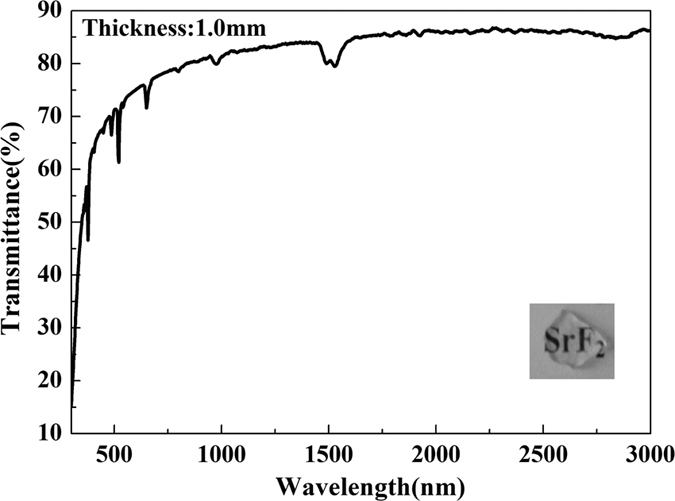
Transmittance spectrum of the Er^3+^-doped GC containing SrF_2_ (1-mm thick). The photograph in the inset shows transparency of the GC.

The transmittance is a very important factor in the assessment of the optical performance of transparent GCs. Based on the Rayleigh-Ganz particle scattering theory^24^, the grain size in a large number of GCs is limited to less than the corresponding wavelength to maintain high transparency. Generally, GCs are translucent or even opaque when the size of the grains reaches the micron level because of strong light scattering^9^, which indicates a poor potential for optical applications. Interestingly, a transparent Er^3+^-doped GC containing micron-sized SrF_2_ crystals was obtained by the abovementioned liquid-phase sintering route, as shown in the inset of Fig. 3, which contained crystals with diameters of approximately 15 μm. It can be seen that the average transmittance of the GC is about 75% in the visible region and more than 85% in the near-IR region. The transmittance is ~83% around 2.7 μm. The high transparency of the samples is attributed to the matching of the refractive index between the SrF_2_ crystals and PG. In order to examine this hypothesis, the refractive index of the SrF_2_ single crystal and PG was measured as 1.442 and 1.447, respectively. This result agrees with the conclusion from the in-line transmittance spectra analysis. The stretching vibration of the free [OH^−^] groups shows a strong absorption band at 2.5–3.0 μm, which affects the 2.7 μm emissions of Er^3+^ ions^25^,^26^. A small [OH^–^] group absorption peak (~2.85 μm) is observed in GCs, indicating that the samples possess a strong ability for water removal. Owing to the similar ionic sizes of F^−^ and OH^–^ ions and the isoelectronic properties, F^−^ ions can efficiently replace OH^−^ ions during liquid-phase sintering^27^.

**Figure 4:**
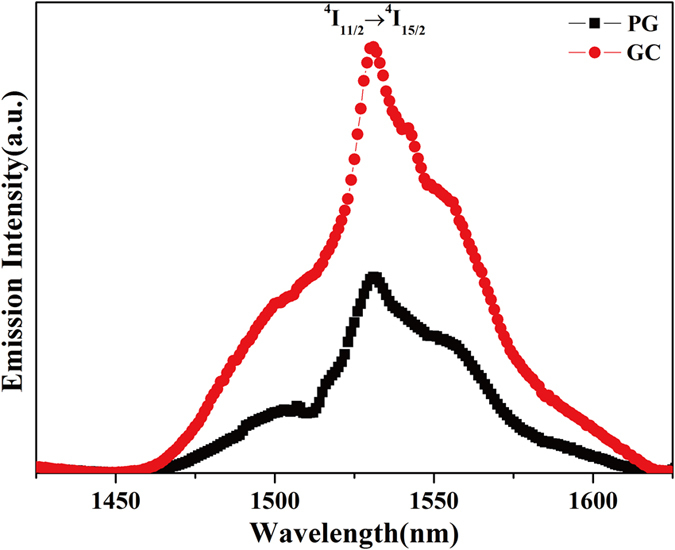
Fluorescence spectra of samples at 1.53 μm, excited at 980 nm.

Wei^6^ and Fan^9^ have pointed out that the efficient 2.7 μm emissions of Er^3+^-doped GCs containing fluoride crystals is attributed to the Er^3+^ ions entering the fluoride crystal lattices. Thus, Er^3+^ ions in crystals embedded into the glass matrix play a crucial role in mid-infrared emissions. In order to confirm that the Er^3+^ ions are incorporated into the SrF_2_ lattices during the GC preparation process, the fluorescence spectra at 1.53 μm was investigated, as presented in Fig. 4. Under 980 nm laser excitation, both PG and GC show 1.53 μm emission bands attributed to the transition of Er^3+^:^4^F_13/2_→^4^I_15/2_^28^. Notably, the GC exhibits stronger 1.53 μm emissions than the PG, and the integrated intensity of the GC is about 2.15 times higher than that of the PG. Decay lifetimes of the Er^3+^:^4^S_3/2_, ^2^H_11/2,_^4^F_9/2_, and ^4^I_13/2_ level in the prepared samples excited at 980 nm were also recorded, as shown in Table.1. Obviously, the decay lifetimes originated from the Er^3+^:^4^S_3/2_, ^2^H_11/2,_^4^F_9/2_, and ^4^I_13/2_ level in the GC are much longer than that of PG. The changes observed in both the photoluminescence (PL) spectra and PL lifetimes indicate that the doped Er^3+^ ions are embedded in the low-phonon-energy SrF_2_ crystals during liquid-phase sintering.

**Figure 5:**
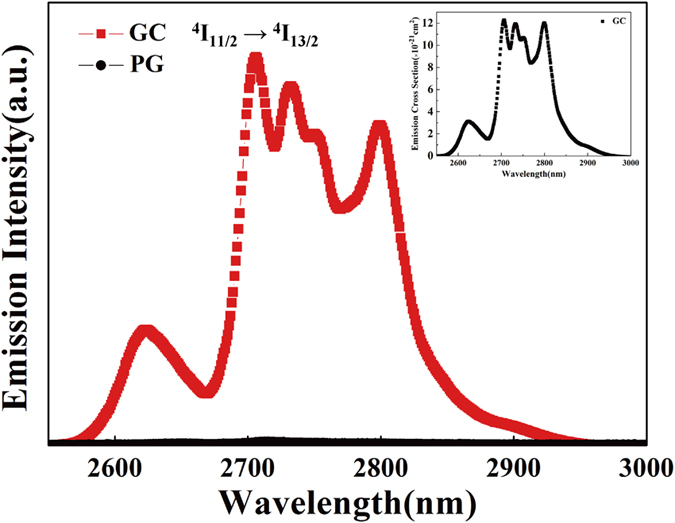
Mid-infrared fluorescence spectra of samples excited at 980 nm. The inset shows the emission cross section at 2.7 μm.

The mid-infrared emissions of the Er^3+^-doped PG and Er^3+^-doped GC containing SrF_2_ crystals were investigated, as presented in Fig. 5. An intense fluorescence around 2.7 μm is observed in GC and no mid-infrared emissions are generated in PG, indicating that the mid-infrared emissions result from the Er^3+^ ions in the low-phonon-energy SrF_2_ distributed in the glass matrix rather than the Er^3+^ ions in the glass matrix. Meanwhile, the ^4^I_11/2_ → ^4^I_13/2_ transition shows an evident Stark splitting in the GC, implying that Er^3+^ ions are incorporated into the SrF_2_ lattices and the ^4^I_11/2_ → ^4^I_13/2_ transition is influenced by the crystal field of SrF_2_^29^,^30^. Mid-infrared fluorescence quenching of Er^3+^ in PG results from strong nonradiative decay due to multiphonon relaxation^9^, which is attributed to a large amount of high-phonon-energy phosphate in PG. To estimate the 2.7 μm emission properties, the emission cross section of GC is calculated as follows^31^:


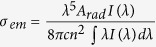


where A_rad_ is the radiative transition probability at 2.7 μm and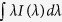 is the normalized line-shape function of the emission spectrum. The inset in Fig. 5 shows the emission cross section of the GC. We can see that the maximum emission cross section is more than 1.2 × 10^−20^ cm^2^, which is obviously larger than that of the Er^3+^-doped oxyfluoride tellurite glass (4.5 × 10^−21^ cm^2^)^32^, ZBLAN glass (5.7 × 10^−21 ^cm^2^)^23^, FP glass (6.57 × 10^−21^ cm^2^)^23^, germanate glass (8.44 × 10^−21^ cm^2^)^27^, bismuthate glass (9.53 × 10^−21^ cm^2^)^11^, and GCs containing NaYF_4_ (7.05 ± 0.25 × 10^−21^ cm^2^)^6^. A large emission cross section indicates that the GC would be a promising candidate for IR lasers and amplifiers.

**Figure 6:**
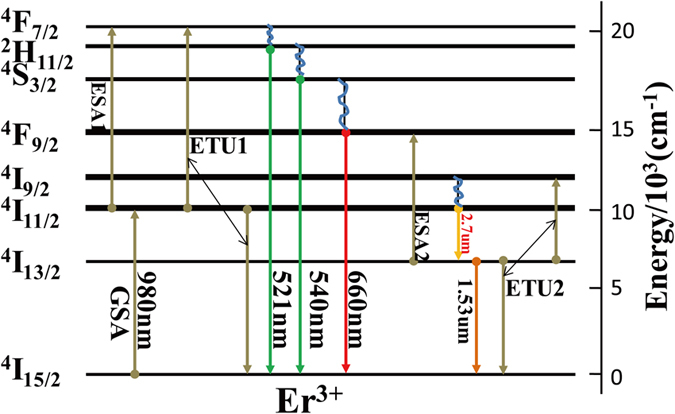
Energy level diagram and energy transfer processes of Er^3+^ ions.

To illustrate the 2.7 μm emission behavior, the energy level diagram and energy transfer mechanism of Er^3+^ ions, as shown in Fig. 6, have been proposed. Electrons at the ground state absorb energy and jump into the ^4^I_11/2_ level via ground state absorption (GSA) upon excitation at 980 nm. Electrons in the excited state ^4^I_11/2_ will relax in two ways. Electrons cycle back to ^4^I_13/2_ by radiative or nonradiative relaxation. Radiative transitions of the electrons generate the 2.7 μm emissions, while the nonradiative transitions cause fluorescence quenching. Meanwhile, electrons in the ^4^I_13/2_ level can further relax to the ground state, emitting at 1.53 μm. Furthermore, the excited state absorption (ESA2: ^4^I_13/2_ + a phonon → ^4^F_9/2_) and the energy transfer up-conversion (UC) (ETU2: ^4^I_13/2_ + ^4^I_13/2_ → ^4^I_9/2_ + ^4^I_15/2_) process, which are conducive for population reduction of the ^4^I_13/2_ level and improving the 2.7 μm emissions, occur simultaneously in the electrons at the ^4^I_13/2_ level^6^. On the other hand, electrons in the ^4^I_11/2_ level are excited to the ^4^F_7/2_ level by the excited state absorption (ESA1: ^4^I_11/2_ + a phonon → ^4^F_7/2_)^33^ or energy transfer UC (ETU1: ^4^I_11/2_ + ^4^I_11/2_ →  ^4^F_7/2_ + ^4^I_15/2_)^34^. Then, the electrons in the ^4^F_7/2_ level relax nonradiatively to the ^4^S_3/2_, ^2^H_11/2_, or ^4^F_9/2_ level and cycle back to the ground state, generating green or red emissions.

**Figure 7:**
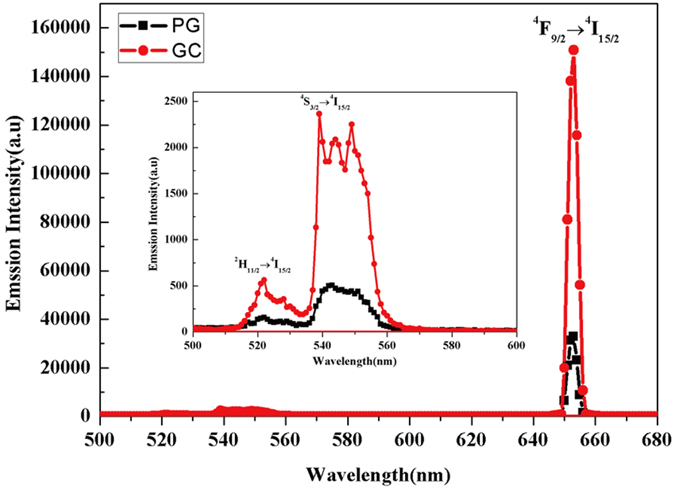
UC emissions spectra of samples excited at 980 nm. The inset shows the UC emissions at 500–600 nm.

Based on the 2.7 μm emission behaviors, the ESA2 process is beneficial for electron accumulation in the ^4^I_13/2_ level and the ^4^F_9/2_ → ^4^I_15/2_ transition. Meanwhile, the ESA2 process can improve the 2.7 μm emissions. Therefore, interactions occur between the UC emissions and the 2.7 μm emissions of the Er^3+^ ions. In order to understand the relationship between the mid-infrared emissions and the UC emissions of the Er^3+^ ions, the UC emissions spectrum of GC and PG is shown in Fig. 7. Under 980 nm laser excitation, the emission peaks at 521, 540, and 653 nm in Fig. 7 are attributed to the transition of Er^3+^:^2^H_11/2_ → ^4^I_15/2_^35^,^4^S_3/2_ → ^4^I_15/2_^35^, and ^4^F_9/2_ → ^4^I_15/2_^36^, respectively. It can be seen that the intensity of the red light emissions is much stronger than that of the green light emissions in PG and GC, which is ascribed to the ESA2 process. It is noted that the GC exhibits a much stronger UC luminescence than the PG, and the integrated intensity of the GC is about 4.5 times higher than that of the PG. Meanwhile, the degree of growth of the red light emissions is larger than the green emissions in the GC. This result indicates that the low-phonon-energy SrF_2_ crystals make the ESA2 process, as well as the ESA1 and EUT1 process, stronger. The UC emissions spectrum indicates that the incorporation of Er^3+^ into the SrF_2_ crystal lattices results in an enhancement of both the UC and mid-infrared emissions.

## Conclusion

Er^3+^-doped transparent GCs containing micron-sized SrF_2_ crystals were successfully prepared using a liquid-phase sintering route. The SrF_2_ crystals were ~15 μm in size and were uniformly distributed throughout the fluorophosphate glass matrix. The GCs achieved average transmittances of 75% in the visible region and more than 85% in the near-IR region by matching the refractive index of the precursor glass with that of the SrF_2_ crystals. The EDS, PL spectra, and PL lifetime results indicate that Er^3+^ ions were incorporated into the SrF_2_ micron-sized crystals. Intense 2.7 μm emissions derived from the transition of Er^3+^: ^4^I_11/2_ → ^4^I_13/2_ were observed upon excitation at 980 nm using a laser diode because of the incorporation of Er^3+^ ions into the low-phonon-energy micron-sized SrF_2_ crystals. The maximum emission cross-section of the Er^3+^:^4^I_11/2_ → ^4^I_13/2_ transition is more than 1.2 × 10^−20^ cm^2^ at 980 nm excitation, implying that there is good potential for fabricating transparent GCs containing micron-sized SrF_2_ crystals with good spectroscopic properties for mid-IR lasers.

## Additional Information

**How to cite this article**: Jiang, Y. *et al*. Er^3+^-doped transparent glass ceramics containing micron-sized SrF^2^ crystals for 2.7 µm emissions. *Sci. Rep*. **6**, 29873; doi: 10.1038/srep29873 (2016).

## Figures and Tables

**Table 1:** Decay lifetimes of the Er^3+^:^4^S_3/2_, ^2^H_11/2,_^4^F_9/2_, and ^4^I_13/2_ levels in the prepared samples excited at 980 nm.

Sample	Lifetime of corresponding wavelength (ms)			
	521 nm	540 nm	653 nm	1530 nm
PG	1.65	1.64	4.23	5.84
GC	3.25	3.14	8.05	11.73
